# Embedding the International Classification of Functioning, Disability and Health in Health Professions Curricula to Enable Interprofessional Education and Collaborative Practice

**DOI:** 10.1177/2382120520933855

**Published:** 2020-08-31

**Authors:** Monica Moran, Jane Bickford, Sarah Barradell, Ingrid Scholten

**Affiliations:** 1 Western Australian Centre for Rural Health, University of Western Australia, Geraldton, WA, Australia; 2 Speech Pathology, Flinders University, Bedford Park, SA, Australia; 3 Physiotherapy, Department of Health Professions, Swinburne University of Technology, Hawthorn, VIC, Australia

**Keywords:** International Classification of Functioning, Disability and Health, ICF, curriculum, interprofessional, education

## Abstract

The World Health Organization’s International Classification of Functioning, Disability and Health (WHO-ICF) is a comprehensive and highly adaptable framework that provides a universal language and shared health concepts to articulate human functioning across the lifespan and from individual to population health settings. It provides a global, biopsychosocial, and holistic structure for conceptualising the human experience of health and health service provision. Consequently, the ICF framework offers hope for a universal map for health service providers that bridges professional, cultural, economic, and geographical variations. While the use of the ICF is typically mandated by health professions accreditation bodies, integration of the ICF in medical and health professional education programmes has been slow. In addition, its potential for scaffolding interprofessional education for collaborative practice has not been maximised. In this Perspective paper, we draw on our extensive experience in developing curricula and teaching within a range of health professions programmes (medicine, occupational therapy, physiotherapy, and speech-language pathology) to provide advice on conceptual, theoretical, and practical dimensions of embedding the ICF framework within curricula to support interprofessional education and collaborative practice.

## Introduction

Interprofessional education for collaborative practice (IPECP) has been posited as a pathway for improving the delivery of complex health services in the 21st century. Its potential to enhance safety, efficiency, and the client experience are well documented.^1^ The World Health Organization (WHO) seminal report, ‘Framework for Action on Interprofessional Education and Collaborative Practice’^2^ provides further support for the integration of shared international frameworks, taxonomies, and communication strategies to provide holistic and team-based health and social services worldwide.

**Figure 1.**
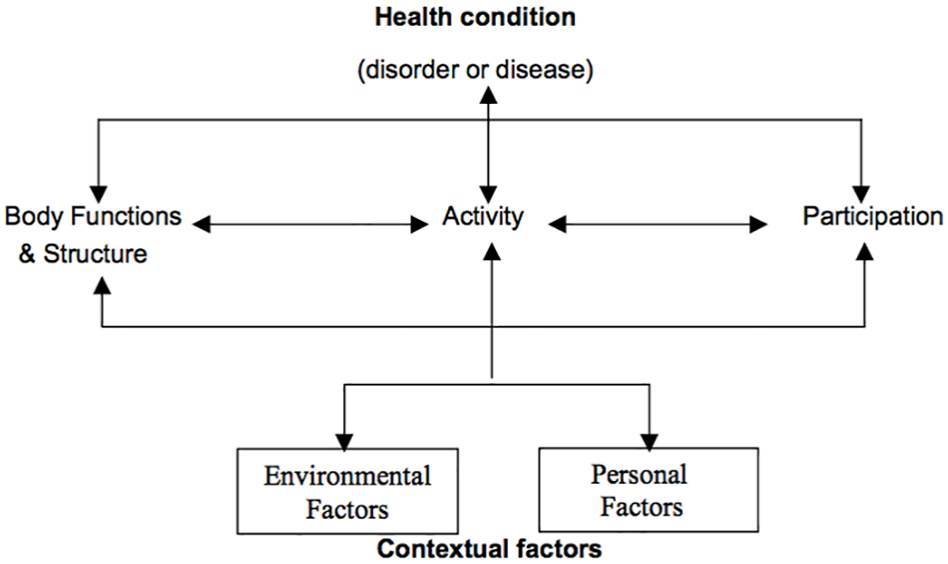
The ICF Framework^5(p9)^ Permission granted; World Health Organization Press – (Permissions Management, Licencing and Reprint Rights).

The International Classification of Functioning, Disability, and Health (ICF) is a comprehensive classification system with a stated aim of establishing a common health language to articulate human functioning across the lifespan and from individual to population health settings.^3^ The WHO has recommended utilisation of the ICF in many jurisdictions, and there is widespread acceptance of the ICF framework^4^ (depicted in Figure 1). However, uptake of the ICF framework as an educational and clinical tool to inform health and social practice across the spectrum of health professions has been slow and patchy in health curricula globally.^6-15^

Despite the limited evidence, there is increasing attention from educators in the application of the ICF as a learning framework to scaffold IPECP.^16^ Stimulated by the international interest in IPECP, a number of jurisdictions (e.g. Canada and USA) have developed agreed learning outcomes and competencies to guide interprofessional learning of health personnel. These capabilities articulate with multiple ICF values and domains, providing opportunities to link learning using the ICF framework within health curricula to achieve IPECP learning outcomes.

Table 1 provides a brief summary of published IPECP learning outcomes and interprofessional competency frameworks and explores the links between the ICF framework and IPECP, and the possible opportunities for the development of interprofessional learning opportunities and attainment of learning outcomes. Broadly, there are shared values, language, and methods between these 2 approaches; each is person/population centred, has a focus on equality and social justice and is informed by evidence-based practice.

**Table 1.** Interprofessional competency frameworks.

Source	Interprofessional learning outcomes or competencies	Synergies with ICF domains
Learning outcomes for interprofessional education (IPE): Literature review and synthesis^17^ This review (a supporting document of the 2010 WHO report) identified 6 major themes that classified the interprofessional learning outcomes reported in 88 published papers	• Teamwork • Roles/responsibilities • Communication • Learning/reflection • The patient • Ethics/attitudes	Person/population centred Shared terminology/language Shared collaborative goal setting Opportunities to explore own scope of practice Opportunities to explore shared scopes of practice Intersectoral collaborations Shared values Informed by evidence-based practice Focus on equality and social justice Provides standardised ways of understanding and articulating patient functioning
Canadian Interprofessional Health Collaborative: National Interprofessional Competency Framework^18^ This national framework was one of the first to be published. It was developed by a national collaborative of Canadian health researchers to inform interprofessional education and collaborative practice across Health Canada. The authors generated 6 core competencies for interprofessional education and collaborative practice	• Interprofessional communication • Patient/client/family/community-centred care • Role clarification • Team functioning • Collaborative leadership • Interprofessional conflict resolution	
Core competencies for interprofessional collaborative practice: 2016 update^19^ This document was developed by a national team (the Interprofessional Education Collaborative) representing 15 associations of health professionals in the United States. Its focus is on informing health professional education, accreditation standards and formation of Faculty Development Institutes. These recent revisions include a greater focus on interprofessional health service delivery at a population level	• Values/ethics – work with other professions to maintain a climate of mutual respect and shared values • Roles/responsibilities – use knowledge of own professional role and of other professionals to assess and address health needs of patients and promote and advance the health of populations • Interprofessional communication – communicate with patients, families, communities and professionals in a responsive and responsible manner that supports a team approach to promotion, maintenance of health, and prevention and treatment of disease • Teams and teamwork – apply relationship-building values and principles of team dynamics to perform team roles to plan, deliver, and evaluate patient/population-centred care and population health programmes and policies that are safe, timely, efficient, effective, and equitable	

Abbreviations: ICF, International Classification of Functioning, Disability, and Health; IPE, interprofessional education; WHO, World Health Organization.

## The Case for Introducing another Framework into Crowded Curricula

The barriers and challenges to implementing IPECP are well articulated in the literature.^20^ Many barriers, such as over-reliance on uniprofessional learning modules, lack of shared language and practice frameworks across the professions, and limited authentic interprofesssional learning opportunities, could be mitigated by incorporating the ICF framework into health professional curricula.^16^ Access to a common set of meanings and language for shared communication is an essential condition for interprofessional education and collaboration.

The theoretical similarities underpinning the ICF and IPECP (biopsychosocial, socioecological, person-centred, and evidence-based practice) would allow ICF to enhance IPECP activation. Both the ICF and IPECP support the incorporation of strategic global solutions to major international health challenges. Policy-based synergies include a focus on integrated team-based service delivery, self-management, consumer-led health care, and health literacy. Practical synergies between ICF and IPECP allow us to tap into a range of resources, including the ICF agreed shared language and ICF-informed clinical tools, assessments and evaluations that can support interprofessional education and team-based care, for example, Australian Therapy Outcome Measures (AusTOMs)^21^ and Canadian Occupational Performance Measure (COPM).^22^

In the next section of this article, we describe the process for developing an ICF-informed IPE curriculum. We then review a number of freely available, practical learning resources and activities, informed by the ICF and with international application that can be used at various points on the student learning journey.

## Embedding the ICF in Health Professions Curricula

There are three stages of designing, building, and evaluating the implementation of an ICF-informed curriculum to support IPECP and ultimately improve health care practice.

### Stage 1: developmental and integrated curriculum design

Developmentally informed curricula are designed to meet students’ learning needs over their educational trajectory and consider contextual, individual, and institutional drivers.^23^ Curriculum design must start with the identification of where we want students to finish, that is, the learning outcomes/competencies we want them to achieve. Identifying these end points requires careful and comprehensive consultation with many stakeholders. Steps for this work include:

1. Identify curriculum stakeholders (colleagues, peers, senior leaders, accreditation gate-keepers, students, and service-users). Together decide on the learning gaps or priorities that need to be addressed and the anticipated outcomes.
2. Map existing curricula to identify areas of overlap/common interest where interprofessional learning outcomes can be achieved and ICF content overlaid to achieve final learning outcomes.
3. Articulate the graduate outcomes/capabilities across professions (e.g., person-centred care, effective team membership and leadership, ethical practice, and effective communication).
4. Generate a list of concepts that the ICF can add to existing health professional education with a focus on enhancing the attainment of the graduate outcomes/capabilities already identified, and communicate to educational stakeholders.
5. Use developmental curriculum design to facilitate students’ learning about the ICF in ways that contribute to their growth as contemporary health care professionals and allow translation for use in practice.^24,25^
6. Build the interprofessional curriculum development team, including educational and student stakeholder representatives and wherever possible service-users.

### Stage 2: build learning experiences and programmatic assessment across the curriculum

At its most practical-level curriculum development involves designing and implementing a set of learning experiences, modules, materials, and assessments across the programme. Assessment processes with elements of programmatic assessment such a lower stakes assessment, and a combination of formative and summative processes should be embedded throughout.^26^

1. Working with key stakeholders, confirm ICF learning outcomes which may be in clusters depending on students’ developmental level (e.g, outcomes for early career students may focus on understanding new concepts, whereas those for more advanced students might focus on capacity to analyse and integrate the ICF into practice). Learning outcomes should be integrated and flow developmentally from simple to complex and beginner to advanced. These processes are well described in integrated curriculum programmes such as the University of British Columbia (UBC) Model for Interprofessional Education^24^ and University of Washington (UW) Health Sciences Curricular Framework for Interprofessional Education^25^ where students complete exposure-type learning activities early in their programmes, progressing to immersion-based learning as their skills develop, and activities focused on mastery of practice as they prepare to transition to their professional lives. Models such as these are based on the assumption that there are ‘optimal learning times for health and human services students (and practitioners) depending upon their stage of development as professionals in their respective disciplines and their readiness to learn and develop new perspectives on professional interaction’.^24(p9)^
2. Using learning outcomes as an overarching guide, generate a set of learning experiences that are informed by developmentally informed taxonomies such as Bloom’s Taxonomy^27^ or Fink’s Significant Learning Experiences.^28^ These cognitive taxonomies provide practical guidance on designing and articulating learning outcomes and experiences in a developmental trajectory. For example, early learning outcomes (such as targeted within exposure experiences) may focus on the development of foundational knowledge and are articulated with terms such as ‘understand’ and ‘identify’, while learning outcomes further along the developmental trajectory may focus on the achievement of learning outcomes related to more demanding immersive learning experiences such as ‘Integrate’, ‘collaborate,’ and ‘create’.^28^
3. Generate both formative and summative assessment activities that will allow students and their educators to identify attainment of developmental learning outcomes across the programme. Assessment feedback must provide meaningful information to drive continued learning.^29^
4. Generate active learning opportunities including wherever possible field-based learning and working with consumers as educators.
5. Embed ICF language in all learning materials.

### Stage 3: research and evaluation

Design an evaluation plan to ensure that the development, integration, and results of the ICF curriculum can be clearly measured.

1. With key stakeholders, co-design an evaluation plan that includes developmental, cross-sectional, and longitudinal evaluation elements. Developmental evaluation involves processes to assess how a programme is functioning on an ongoing basis and the introduction of changes to address performance ‘on the run’. Examples might involve weekly team meetings to explore progress and generate ways to overcome any identified barriers. Cross-sectional evaluation can occur at designated time-points, for example, at mid-term or end of semester, to evaluate outcome attainment and stakeholder satisfaction. Longitudinal evaluation looks at the achievement of outcomes over time and how these achievements have contributed to graduate performance, destination, and longer term contribution. As a minimum, incorporate an annual review that includes feedback on the student experience and institute changes and modifications as part of a cyclic quality-improvement approach.
2. Facilitate regular team meetings to reflect on curriculum processes and progress.
3. Engage with other programmes running ICF embedded curricula to allow for collaboration, benchmarking, evaluation, and research opportunities.

This process of designing, building, and evaluating an ICF-informed curriculum relies on strong communication skills and collaboration with stakeholders – also key to working effectively in health care.

## Educational Resources

Introducing the ICF as an additional framework to already busy curricula is a challenge for health educators and students alike. While the ICF informs many of the practice theories and models that drive individual health professions, learners can struggle to see how the ICF superimposes on their own profession-specific learning schema, practice theories, and models. Ways to reveal these synergies between the ICF, profession-specific and interprofessional content are evolving through active learning activities. Tools and conceptual models of various levels of complexity and pertaining to different health professions have been designed to facilitate development of ICF application skills to improve practice, including decision-making frameworks to enhance students’ reflection and reasoning.^30,31^ Here, we review a number of ICF-informed tools and models we have successfully used in our educational practices.

### Example 1: ICF functional profile

**Figure 2.**
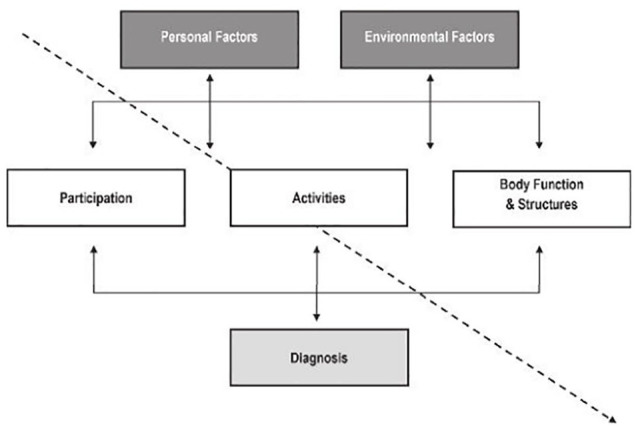
The inverted ICF model.^31(p61)^

**Figure 3.**
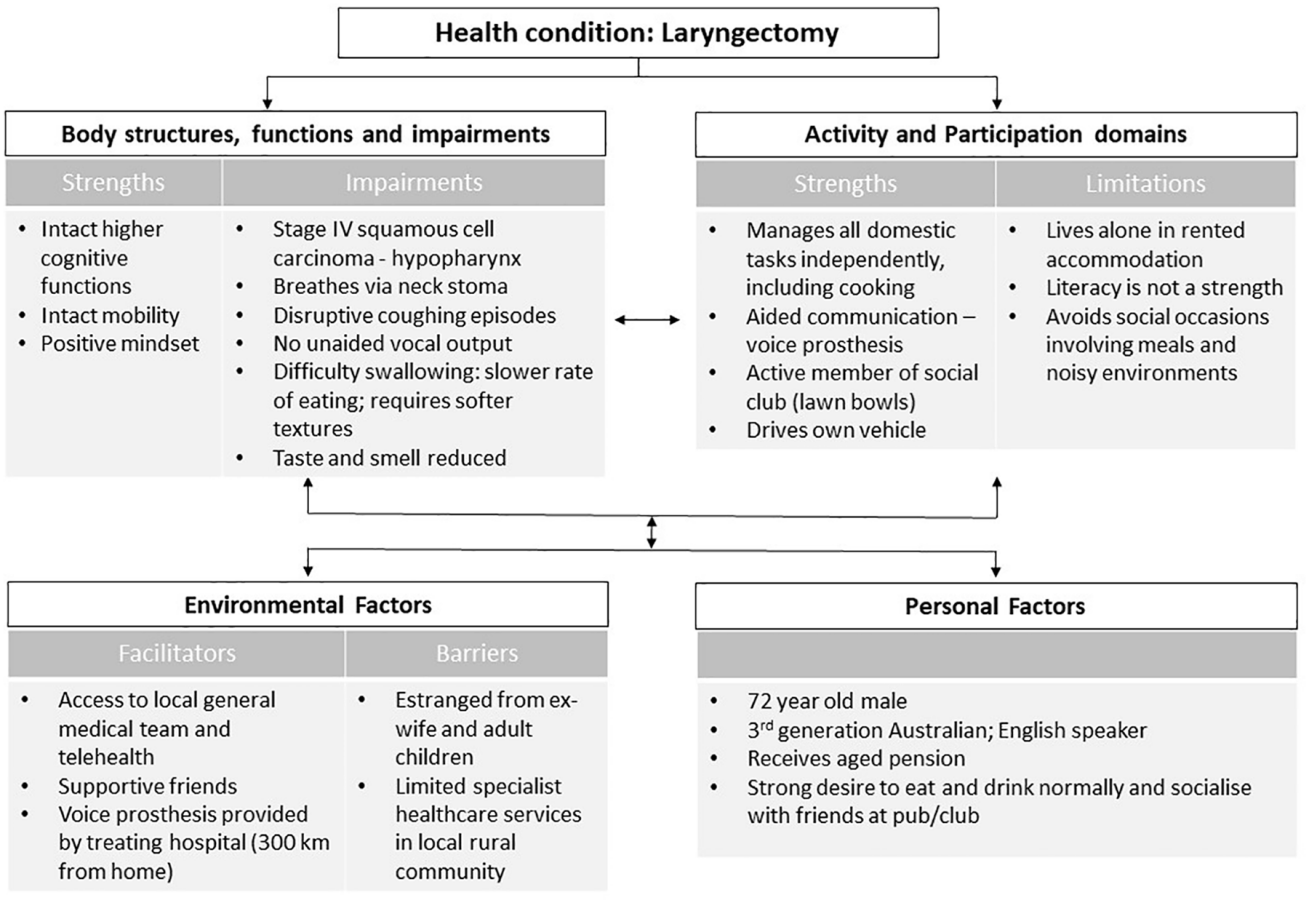
Example of a completed ICF functional profile for a person following total laryngectomy.

The ICF conceptual framework itself is a tool that is often used to document and guide practice.^32^ An ICF functional profile can help direct practitioners’ attention to areas beyond the health condition and related body functions and structures, including identifying barriers and enablers to participation, to help plan intervention that optimises functioning and well-being and track changes related to a health condition’s natural history, interventions provided, or environmental adjustments made.^32^ Several suggestions exist for how to create a profile according to the ICF components, typically using ordinary language, although ICF coding may also be included. Simon and Kraus de Camargo^31^ describe when it might be helpful to invert the model to ‘read it’ from a different direction, in keeping with a person-centred approach to care and the ICF model’s use of bidirectional arrows throughout (Figure 2). Please note that the connecting arrows in this diagram are not consistent with WHO depictions of the model; additionally, arrows between participation and activities, and between activities and body functions and structures, are missing from this published figure; they should all be reinstated appropriately when using it with students). This can help highlight the influence of contextual factors on the service-user’s condition and level of functioning. Simon and Kraus de Camargo^31^ describe how to create an ICF functional profile which includes strengths and limitations for each component. Figure 3 depicts the profile for an individual following laryngectomy.^33^ The Physical Therapy Clinical Reasoning and Reflection Tool (PT-CRT)^30^ takes a similar approach to creating a profile. It is one component of a detailed reflection and discussion tool designed to be used, either partially or in full, within a client management model. While similar, the Rehabilitation Problem-Solving Form^34^ includes space for the service-user’s perspective, such as their symptoms and experiences, as well as the practitioners’ evaluation, including assessment findings and ICF classifications.

The use of case studies, whether informally to illustrate a point, or formally in case-based or problem-based learning curricula, is a popular way to educate health professional students. Case method is used globally at undergraduate and graduate educational levels, employing inquiry-based learning to convey relevance and prepare students for clinical practice.^35^ Students enjoy case method, believing that it improves their learning; educators like it because it captivates and motivate students.^36^ In fact, case method has been shown to ‘enhance clinical knowledge, improve teamwork, improve clinical skills, improve practice behavior, and improve patient outcomes’.^35(p47)^ A systematic and extensive use of case studies provides myriad opportunities for students to engage with the ICF framework, to consider the individual, their functioning, preferences, and facilitators and barriers to their participation in life. Case studies can involve student creation of an ICF functional profile, initially in a guided way, such as when learning disciplinary approaches to assessment. Later, ICF profiles might be incorporated at summary points within or on conclusion of a case. They can also be used to help embed the ICF framework into routine tasks, such as writing reports, determining appropriate intervention, or conducting case presentations.

### Example 2: the F-words knowledge hub

**Figure 4.**
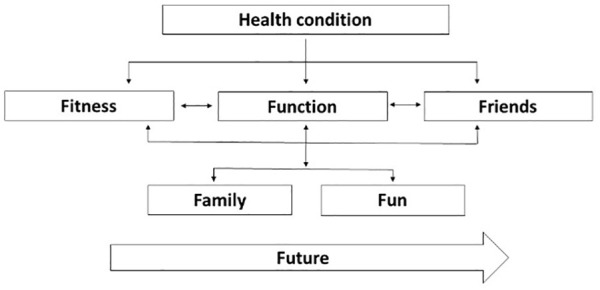
ICF F-words.^37^

Rosenbaum and Gorter^37^ describe how the ICF dimensions can be operationalised in the field of childhood disability in a series of ideas called ‘F-words’. These words – function, family factors, fitness, fun, friendships, and future – capture the priorities for working with children and have been further simplified to map directly to the ICF framework (Figure 4). A word of caution, however. While Rosenbaum and Gorter’s use of the term ‘function’ to encapsulate activities may make the ICF more accessible to service-users, it has the potential to confuse students because the term ‘function’ is actually linked to the body perspective in the ICF, rather than being associated with activities. Nonetheless, this is an engaging resource with considerable merit if used carefully. The development of the F-words was informed by sustained and comprehensive research with practitioners, families and children and has evolved into the F-words Knowledge Hub as part of the CanChild Research centre at McMaster University in Canada (https://www.canchild.ca/en/research-in-practice/f-words-in-childhood-disability). Researchers have developed a range of resources including child-centred assessments and intervention protocols that are freely available for students and educators to download for clinical and educational purposes.

Experience suggests that students can make the most of the resources if they have brief revision of the ICF domains before using specific tools in an interprofessional practical learning environment. Students completing professional practice in a paediatrics environment where they have an opportunity to use the F-Words Agreement will learn about child/family-centred approaches authentically by tapping into the priorities of the child using age appropriate language. The F-Words Goal Sheet allows students to work with the child and family to generate shared priorities for the child that can be supported by the student team. The key aspect is that goals reflect the child’s priorities for function (informed by the ICF). The F-Word team at the CanChild Research Centre are currently translating the resources into a number of languages so that they are freely available for use across many countries and cultures.

### Example 3: contexts of participation critical thinking tool

The engaging Contexts of Participation critical thinking tool^38^ was informed by the ICF and the social determinants of health to help students appreciate a social model of care and understand how participation can be enabled or hindered by both individual and environmental factors. Ghul and Marsh^38^ described application of the tool within an occupational therapy programme, where it is used by students to analyse narratives presented by service-users and carers, the students’ own stories, or those portrayed in the media, including newspapers, film, or novels. The tool helps students become more aware of power relationships and related underlying assumptions and to appreciate the sometimes-obscure effects on participation.

**Figure 5.**
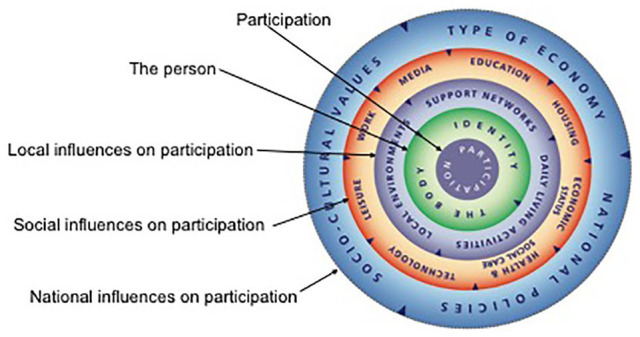
Contexts of participation – The Critical Thinking Tool^38^ Permission granted; Canterbury Christ Church University.

The simple downloadable tool (see Figure 5) is constructed from 5 centrally secured concentric cardboard rings. The innermost focus is *participation*, with the outer rings denoting various influences – personal (identity; the body), local (support networks; daily living activities; and local environments), social (education; housing; economic status; health and social care; technology; leisure; work; and media), and national (type of economy; national policies; and sociocultural values). The central placement of *participation* helps convey the reciprocal interaction between the person and their environment; the original placement of *health and well-being* in this location meant that students often failed to appreciate that it is a product of participation.^38^ Physical interaction with the wheel (cutting out the template and turning the circles to create different alignments when exploring issues) seems to aid engagement with the process and ‘make creative and critical links between the categories’.^38(p9)^

The tool can also be used for purposes other than analysing narratives. For example, it helps students to frame questions, such as when learning to take a client history, for detailed qualitative interviews, or during classroom presentations by service-users. Students can become acquainted with the tool by comparing it with the ICF framework, considering whether there are subtle preferences afforded by either model. Achieving the more ambitious goal of creating a transformative educational programme where students become change agents operating within a social model of care is likely to require a clear focus on curriculum redesign, based around the concept that health and wellness are facilitated by participation. The Contexts of Participation tool can assist educators to consciously address the many features detailed on each circle of the wheel to ensure breadth and depth in narratives/scenarios created for student exploration – that is, to ‘cover the wheel’.

Students require assistance to optimise the benefits of using this analytical tool. At a minimum, students will need to be supported through the critical thinking process, with prompts, a list of steps or suggested questions. These help students to organise the information presented within the narrative being considered, structure their reasoning, identify underlying assumptions, evaluate the arguments made, remain flexible in their thinking, and reflect on what has been learned.^38^

### Example 4: the MAGPIE model for interprofessional case-based teaching

The MAGPIE model was originally developed by a team of health professionals working in Australian community rehabilitation settings as part of a workforce redesign project.^39^ The MAGPIE acronym stands for the following clinical team functions: Meet, Assess, Goal-Set, Plan, Implement and Evaluate. The model has since been adapted by health educators as a framework for interprofessional education.^40,41^

The MAGPIE model describes a process to support interprofessional team-based case management. It is informed by the ICF framework and is underpinned by principles of client-centred goal setting, interprofessional collaboration, and holistic care.^40^ It can be used in the classroom as students prepare for placements or as a practice-based tool to guide team-based learning in health care settings. Each stage of the model provides a variety of learning opportunities to build professional and interprofessional skills. The model can be used in an integrated format to guide student learning in a team case conference activity. It can also be deconstructed to allow the development of specific learning and skills development activities that, when combined, allow students to work together collaboratively. Table 2 outlines some of the learning activities that can be designed using the MAGPIE model.

**Table 2.** Suggested learning activities based on the MAGPIE model.^39^

MAGPIE model	Team member function	Examples of learning activities for tutorial or practice locations, as relevant
**M**eet	• Obtain service-user details • Establish rapport • Identify cultural issues • Explain service • Explain rights and responsibilities • Gain consent • Observe environment	• Appraise referral or case-notes and consent procedures as a group activity • Meet service-users (in classroom context). Students use the Contexts of Participation tool^38^ to plan questions they might ask the presenters • Meet new clients – for example, in role plays, with standardised patients or new clients in practice settings. Prepare for the meeting – obtain transcultural information and resources • Explain service and team roles to service-users using role play • Cue students to observe practice setting (environment)
**A**ssess	Ascertain the following: • Impact of illness/injury factors • Impairment of body function and structure • Activity limitations • Participation restrictions • Personal factors • Environmental factors	• Use ICF core sets for different conditions and settings (such as acute care; postacute rehabilitation). See ICF Research Branch for information on Core Sets; ICF Documentation Tools and Rehab-Cycle®. e.g., ICF Assessment Sheet; (https://www.icf-research-branch.org/) • Develop an ICF functional profile for service-user (e.g., within a case study) • Use F-words Knowledge Hub resources to establish service-user’s preferences (fitness, function, friends, family, fun, and future) • Apply the Contexts of Participation tool^38^ to given narratives to explore a range of environmental factors impacting on participation (e.g., case study; newspaper report; scenario with cultural implications) • Compare relevant intraprofessional assessment tools against the ICF domains; Research how the ICF has been mapped against various outcome measures in a specific practice area • Apply the Canadian Occupational Performance Measure (COPM) within a case study (http://www.thecopm.ca/learn/) • Watch client assessment video in a home setting and audit the environment • Conduct joint assessments with other students • Become familiar with various shared assessment tools
**G**oal-set	Establish: • Collaborative alliance • Short-term goals • Long-term goals • Family/community goals	• Observe collaborative goal-setting sessions with experts and clients and plan intervention (see below) • ICF case studies (https://www.icf-casestudies.org/case-studies) • Practise using Talking Mats^42^, and Activity Card Sort^43^ tools to explore service-user priorities
**P**lan	• Analyse facilitators and barriers • Identify strategies to manage/minimise barriers and activate facilitators • Integrate best available evidence regarding values-based practice, intervention, and management	• Within groups, synthesise an intervention plan using assessment data and/or video of goal-setting session with the research evidence of best practice • Identify the care priorities of different professions and develop an holistic plan incorporating a sequence of prioritised strategies and how they will be facilitated or managed to overcome barriers • Determine sequencing of interventions based on contextual factors (access to resources, treatment demands, etc.)
**I**mplement	Apply the following: • Discipline-specific interventions • Interprofessional interventions • Case management skills • Client advocacy • Education for self-management • Health literacy • Whole of community interventions • Referral to other services • Discharge planning • Long term follow-up	• Practise delivery of various interventions – and provide formative and summative feedback, including peer feedback - Include ideas specifically related to improving participation • Reflect on how to empower clients to self-manage • Practise negotiating with clients in roleplays or practice settings • Learn how to run groups and attend to group dynamics • Become familiar with and utilise community resources • Manage client on continuum of care – short term to long term • Learn documentation skills, protocols, and professional terminology, for example, ICF Intervention Table – to record team members’ contributions to treatment
**E**valuation	Assess the following: • Goal attainment • Quality of life • Activity participation • Environmental access	Conduct the following: • Review intervention outcomes in practice settings or in roleplay, for example, ICF Evaluation Display • Track performance over time using appropriate tools (in case studies or simulations, where information about service-user’s performance or attitudes is provided, students may complete relevant forms or interpret data provided) - e.g., AusTOMs; COPM; FIM; WHOQOL • Home and community visits • Post-discharge interviews • Invite consumer perspectives - Discuss and develop methods of measuring outcomes from the perspective of the client, practitioner, family, and health service. • Benchmark with other services

Abbreviations: COPM, Canadian Occupational Performance Measure; FIM, Functional Independance Measure; ICF, International Classification of Functioning, Disability and Health; MAGPIE, Meet, Assess, Goal-Set, Plan, Implement and Evaluate; WHOQOL, World Health Organisation Quality of Life Assessment.

These four examples can be integrated within health professions curricula to enrich the students’ learning experience.

## Conclusion

Our research and experiences in teaching across multiple health professional programmes gives us confidence that the ICF framework is a valuable educational tool in uniprofessional and interprofessional programmes. The values and principles embedded in the ICF align with those of IPECP, including team-based care, client centeredness, and evidence-based and holistic practice. The ICF framework can be introduced at various stages of a developmental curriculum. There is a wealth of learning resources and tools informed by the ICF available to educators. In this Perspective article, we have introduced a selection that has assisted our own educational practices.
